# The Neuroprotective Effect of Shenmai Injection on Oxidative Stress Injury in PC12 Cells Based on Network Pharmacology

**DOI:** 10.1155/2022/6969740

**Published:** 2022-05-27

**Authors:** Jing Wu, Jiang Wu, Zhonghao Li, Xiaoke Dong, Siyuan Yuan, Jinmin Liu, Le Wang

**Affiliations:** ^1^Beijing University of Chinese Medicine, Beijing 100029, China; ^2^Dongfang Hospital Beijing University of Chinese Medicine, Beijing 100078, China; ^3^Beijing Daxing District Hospital of Integrated Chinese and Western Medicine, Beijing 102600, China

## Abstract

**Background:**

Shenmai injection (SMI) has been used in the treatment of cerebrovascular diseases and cardiovascular diseases. However, the underlying mechanism of SMI for neuroprotection after acute ischemic stroke (AIS) remains unclear. This study aimed to explore the potential molecular mechanism of SMI in treating reperfusion injury after AIS and its protective effect on PC12 cells against oxidative stress through in vitro experiments based on network pharmacological predictions.

**Methods:**

The network pharmacology method was used to collect the compounds in SMI and AIS damage targets, construct the “drug-disease” target interaction network diagram, screen the core targets, and predict the potential mechanism of SMI treatment of AIS. In addition, the oxidative stress model of PC12 cells was induced by H_2_O_2_ to evaluate the neuroprotective effect and predictive mechanism of SMI on PC12 cells.

**Results:**

A component-targeted disease and functional pathway network showed that 24 components from SMI regulated 77 common targets shared by SMI and AIS. In PC12 cells damaged by H_2_O_2_, SMI increased cell survival, alleviated oxidative stress injury, prevented cell apoptosis, and increased the expression of APJ, AMPK, and p-GSK-3*β*. After Si-APJ silenced APJ expression, the above protective effect of SMI was significantly weakened.

**Conclusion:**

SMI is characterized by multiple components, multiple targets, and multiple pathways and inhibits oxidative stress and alleviates nerve injury induced by H_2_O_2_ through regulating the APJ/AMPK/GSK-3*β* pathway.

## 1. Introduction

AIS is recognized as the most common cerebrovascular disease and a major public health problem [1, 2]. At present, early restoration of blood supply is considered to be the main treatment strategy for AIS [3]. However, the reperfusion process following an ischemia attack may further exacerbate brain damage, which is called cerebral ischemia/reperfusion (I/R) injury [4, 5]. The pathological mechanism of I/R is complex, involving a variety of pathophysiological processes, such as oxidative stress, inflammatory response, neuronal death, and apoptosis [6, 7]. Therefore, treatment based on the mechanisms described above is considered to be a promising strategy for reducing the consequences of stroke and brain I/R injury.

SMI is a herbal injection approved by China's State Food and Drug Administration (CFDA) in 1995 [8]. It is widely used as an organ protective agent in China for the treatment of cerebral infarction, coronary heart disease, and malignant diseases [9–11]. It consists of aqueous extracts of two traditional Chinese medicine (TCM): Red ginseng (Hong Shen) and Ophiopogonis Radix (Mai Dong). Recent studies have shown that SMI has antioxidant activity and can improve cardiac microcirculation by scavenging oxygen free radicals [12, 13]. It attenuates reperfusion injury in H9c2 cells by modulating mitochondrial dynamics [8]. By reducing the generation of ROS and regulating intracellular calcium and inhibiting cell apoptosis, it has a protective effect on cardiac dysfunction and I/R injury [14–16]. However, due to the lack of in-depth molecular biology studies and the complexity of its chemical components, the mechanism of action of SMI remains unclear.

The orphan receptor APJ and its endogenous ligand apelin are widely distributed in the central nervous system and participate in the pathophysiological regulation of some brain diseases, including AIS [17]. There is increasing evidence that the Apelin/APJ system inhibits apoptosis or death and improves behavioral performance through various mechanisms, including inhibition of excitatory toxicity, inflammatory response, endoplasmic reticulum, and oxidative and nitrifying stress; it also regulates autophagy and promotes angiogenesis, thus showing neuroprotective effects [18, 19]. Studies have shown that Apelin can reduce oxidative stress, autophagy, and apoptosis in PC12 cells by activating PI3K and ERKs while reducing the expression of Beclin-1 and LC3-II [20, 21]. Therefore, targeting APJ signaling pathway may have a protective effect on I/R injury.

Network pharmacology combines chemoinformatics, bioinformatics, and network biology to help reveal the complex pharmacological mechanisms of several TCM preparations [22, 23]. It advocates changing the single-target mode of drugs acting on diseases into a multitarget mode [24]. The interaction pathways between diseases and drug targets can be explained by mining core targets, integrating targets, and analyzing core targets at the molecular level of targets, genes, and pathways [25, 26].

In summary, SMI is a multicomponent, multitarget drug that exerts a protective effect against oxidative stress by regulating molecular networks. Therefore, this study aims to explore and verify the intervention mechanism of SMI on oxidative stress damage in I/R through network analysis and cell experiments.

## 2. Materials and Methods

### 2.1. Effective Ingredients and Targets Collection of SMI

Since 2004, the CFDA has promulgated the “Drug Specifications,” requiring all TCM injections to be standardized through chromatographic fingerprints before being marketed [27].

The National Drug Standard (WS3-B-3428-98-2010Z) issued by CFDA includes the revised standard of SMI. Therefore, the main ingredients of SMI have strict quality control. High-performance liquid chromatography (HPLC) fingerprint and pattern recognition technology were used to identify the quality of SMI produced by different manufacturers, and it was found that the components of samples from different manufacturers had great similarities [28]. HPLC fingerprint and pattern recognition technology have previously been used to analyze the SMI components used in this study, and the results showed that its main chemical components include ginsenosides Rb1, Re, and Rg1, ophiopogonins D and D′, and methylophiopogonanones A and B [29].

Then, the ingredients were screened with OL ≥ 0.18 as the standard. In addition, those main therapeutic target which were mentioned in the multiple studies but less than 0.18 still retain. Then, the included compounds were input into Swiss Target Prediction (https://www.swisstargetprediction.ch/) [30] to standardize the target information, and the target with probability ≥0.1 was screened.

### 2.2. AIS Genes Collection

The keyword “acute ischemic stroke” was used to explore the potential targets of diseases in the GeneCards (https://www.genecards.org) [31] and Online Mendelian Inheritance in Man (OMIM) (https://omim.org/) [32] databases. In GeneCards, the higher the score is, the more closely the target is associated with the disease. If there are too many targets, the target whose Relevance Score is greater than 10 is set as the potential target of the disease. After the combination of two database targets, the duplication is deleted to obtain the disease targets.

### 2.3. Network Construction and Analysis

Cytoscape software (Version 3.8.0) [33] was used to visualize the drug component target network. The core target used the Metascape database (https://metascape.org/gp/index.html) [34] for KEGG pathway enrichment analysis, and the results are visualized through the online mapping tool Bioinformatics (https://www.bioinformatics.com.cn/) to study whether Chinese herbal medicine may participate biologically.

### 2.4. Materials

Highly differentiated PC12 cells (ZQ0150) were purchased from Zhongqiao Xinzhou Biotechnology Co., Ltd. (Shanghai, China). SMI (Lot: Z33020018) was purchased from Zhengda Qingchunbao Pharmaceutical Co., Ltd. (Hangzhou, China). One-Step TUNEL Apoptosis Assay Kit (C1089), Lipid Peroxidation MDA Assay Kit (S0131S), Total Superoxide Dismutase (SOD) Assay Kit with WST-8 (S0101S), and Reactive Oxygen Species (ROS) Assay Kit (S0033S) were provided by Beyotime (Shanghai, China). Anti-AMPK alpha (^#^5831), phospho-AMPK*α* (Thr172) (40H9) (^#^2535), anti-GSK-3*β* (^#^9315), and phospho-GSK-3*β* (Ser9) (^#^5558) were purchased from Cell Signaling Technology, Inc. (Beverly, MA, USA). Anti-APJ (20341-1-AP) and CoraLite488-conjugated Affinipure Goat Anti-Rabbit IgG (H + L) (SA00013-2) were purchased from ProteinTech (Wuhan, China).

### 2.5. Cell Culture and Transfection

PC12 cells were cultured with different concentrations of H_2_O_2_ for 24 h, and the 50% inhibitory concentration (IC50) was selected for subsequent experiments. Then, cells were cultured with different concentrations of SMI for 24 h, and the drug concentration with the highest cell survival rate was selected for follow-up study.

Small interference RNA against APJ (Si-APJ) was synthesized by GenePharma Co., Ltd. (Shanghai, China). Before H_2_O_2_, PC12 cells were transfected with Si-APJ or empty vector, respectively. Transfected cells with Lipofectamine 2000 (^#^11668019; ThermoFisher Scientific, Shanghai, China) for 24 h according to the manufacturer's protocols. Then, the cells were harvested for follow-up experiments as indicated.

### 2.6. Cell Viability Measurements

Cell viability was determined by the CCK-8 assay. PC12 cells were cultured in 96-well plates and treated with H_2_O_2_ and SMI, and then CCK-8 solution (10 *μ*L/100 *μ*L medium) was added and incubated for 2 h. The absorbance of culture medium at 450 nm was detected by microplate reader (Bio-Tek, USA).

### 2.7. TUNEL Staining

PC12 cells were seeded into 6-well culture plates. After treatment, cells were fixed with 4% paraformaldehyde for 30 min. Then, 0.3% Triton X-100 PBS was added and incubated for 5 min. After this, it was incubated with TUNEL detection solution at 37°C for 1 h.

### 2.8. Determination of MDA, SOD, and ROS

The activities of MDA, SOD, and ROS in cells were detected to measure the antioxidant capacity of SMI. According to the manufacturer's protocols of the commercial kits, MDA and SOD values were measured by Microplate Reader, and the total cellular protein was measured with the BCA method for standardization. ROS fluorescence was observed under a fluorescence microscope (BX71; Olympus, Tokyo, Japan).

### 2.9. Immunofluorescence Staining

Briefly, PC12 cells were first fixed with 4% paraformaldehyde for 30 min, and then 0.3% Triton X-100 was added to permeabilize for 5 min. After blocking with 5% BSA for 30 min, APJ primary antibody was added and incubated overnight at 4°C. Next, cells were incubated with a secondary antibody at 37°C for 1 h and DAPI for 5 min. Fluorescence images were obtained with a fluorescence microscope.

### 2.10. Western Blotting

Total protein obtained from PC12 cells was quantified using BCA Protein Quantification Kits. Equal amounts of proteins were separated by SDS-PAGE and transferred onto a polyvinylidene fluoride (PVDF) membrane. After being blocked for 1 h, the membranes were incubated with primary and secondary antibodies at room temperature for 2 h and 1 h, respectively.

### 2.11. Statistical Analysis

SPSS 22.0 and GraphPad Prism 8.0.1 were used for statistical analysis and graph making. The Shapiro–Wilk (SW) method was used to test the normal distribution of quantitative data, the mean ± SD was used to describe the normal distribution, one-way analysis of variance (ANOVA) was used to compare the mean of multiple groups of samples, and LSD was used for comparison between two groups. Non-normal distribution was described by median and quartile, and differences between groups were tested by nonparametric test. Significance level of statistical tests was set at 0.05.

## 3. Results

### 3.1. Identification of Common Targets of AIS and SMI

**Figure 1:**
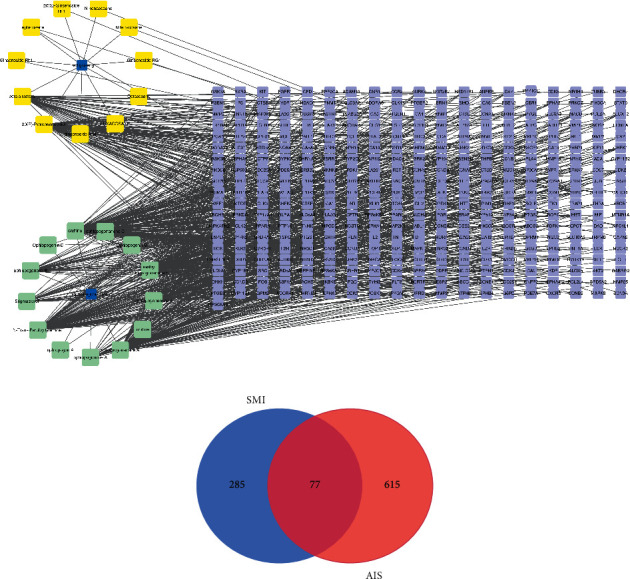
(a) SMI component-target network diagram. (b)The intersection targets of the drug SMI and the disease AIS.

The target data of SMI were obtained through the Swiss Target Prediction. The component-target network was constructed by Cytoscape software, and a network graph of 388 nodes and 861 edges was obtained (Figure 1(a)). Among them, 388 nodes included 11 components of Red Ginseng, 13 components of Ophiopogonis Radix, and 362 targets (Supplementary Table S1). 861 edges represented the relationship between SMI components and targets. Then, the targets of AIS were obtained through the disease-related database, and a total of 692 targets were included (Supplementary Table S2). Among them, 77 targets were shared by both SMI and AIS (Figure 1(b), Supplementary Table S3) and became the focus of our following analysis.

### 3.2. Protein-Protein Interaction Data and KEGG Pathway Enrichment Analysis

**Figure 2:**
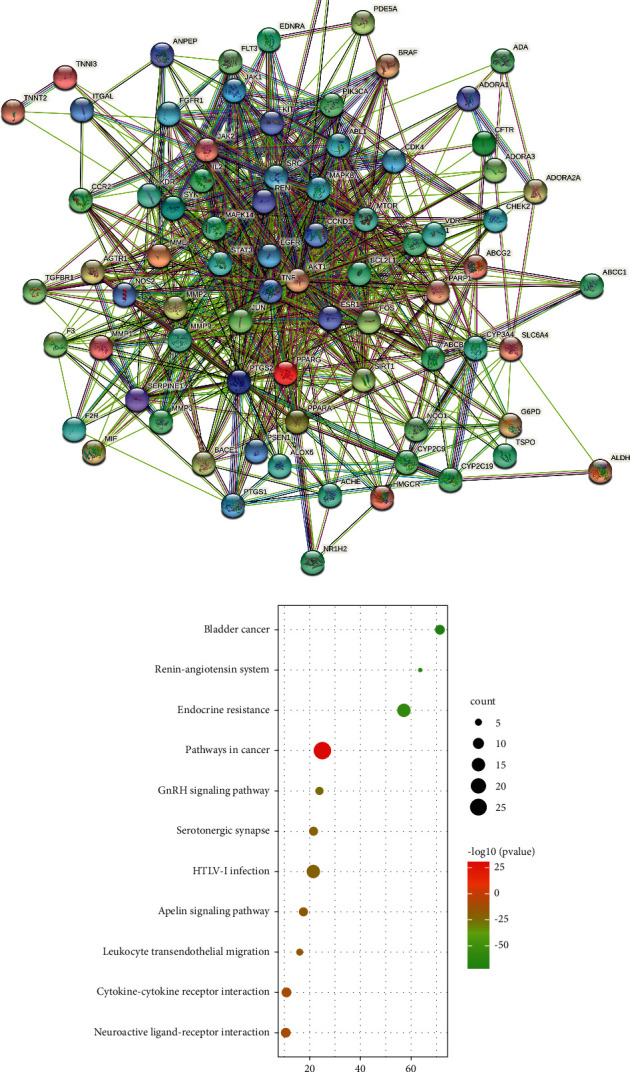
(a) SMI-AIS PPI network. (b) Enrichment map of the KEGG pathway.

77 common SMI and AIS targets were introduced into the STRING11.0 platform to construct the PPI network (Figure 2(a)). According to the rank of degree value, TNF, AKT1, EGFR, SRC, JUN, and STATA3 are the top targets, which are the core targets of SMI for AIS treatment.

Metascape software was used to analyze the KEGG pathway enrichment for core targets, including endocrine resistance, serotonergic synapse, Apelin signaling pathway, and renin-angiotensin system, suggesting that SMI may act on AIS through these pathways. At the same time, the first 10 process pathways were selected by using Bioinformatics to draw the KEGG enrichment analysis bubble map (Figure 2(b)).

### 3.3. Protective Effects of SMI on H_2_O_2_-Induced Cell Death

**Figure 3:**
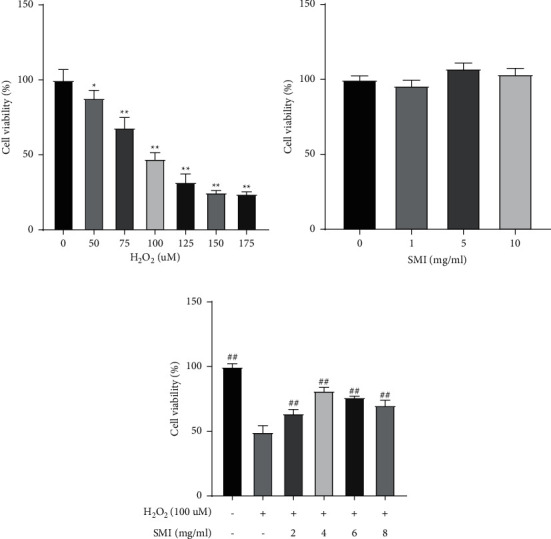
SMI treatment enhances the viability of cells induced by H_2_O_2_. (a, b) Effects of different concentrations of H_2_O_2_ and SMI on the viability of PC12 cells. (c) Effects of treatment with different concentrations of SMI on the viability of PC12 cells induced by H_2_O_2_. ^*∗∗*^*P* < 0.01 vs control group; ^*∗*^*P* < 0.05 vs control group; ^##^*P* < 0.01 vs H_2_O_2_ group.

To assess the effect of SMI on H_2_O_2_-induced oxidative stress, we first measured the effects of different concentrations of H_2_O_2_ and SMI on the viability of PC12 cells by CCK-8 assay. PC12 cells were cultured for 24 h under different conditions, and the results are shown in Figure 3. The higher the H_2_O_2_ concentration, the lower the cell survival rate, while the SMI concentration of 0–10 mg/mL had no significant effect on the cell survival rate. When H_2_O_2_ concentration was 100 µM, the cell survival rate was 45%, which was closest to IC50. Therefore, H_2_O_2_ concentration of 100 µM was used for subsequent experiments. SMI could significantly improve the cell survival rate reduced by H_2_O_2_, and the effect was most significant at 4 mg/mL, which was used for subsequent experiments.

### 3.4. SMI-Ameliorated Cells Injury by Enhancing APJ Level

According to the results of KEGG pathway enrichment analysis in network pharmacology, the APJ level was selected to explore the mechanism of SMI on oxidative stress after AIS.

**Figure 4:**
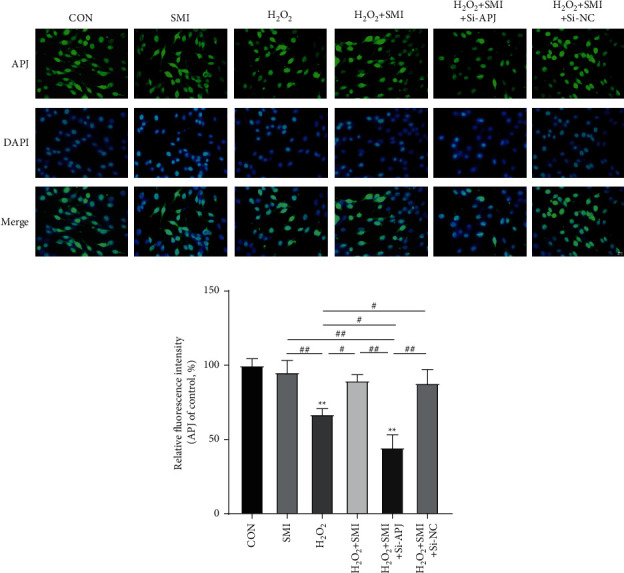
Immunofluorescence images showed the effect of SMI on APJ expression. Scale bar = 20 *μ*M. ^*∗∗*^*P* < 0.01 vs control group; ^#^*P* < 0.05; ^##^*P* < 0.01.

To further confirm the effect of SMI on APJ expression, the immunofluorescence method was used to visually detect APJ content, and Si-APJ was used to silence APJ expression. The changes in APJ fluorescence intensity were observed, as shown in Figure 4. H_2_O_2_ treatment can significantly reduce the fluorescence intensity of APJ. SMI itself has no effect on the fluorescence intensity of APJ but can enhance the fluorescence intensity after H_2_O_2_ treatment. Compared with Si-NC, Si-APJ significantly reversed the effect of SMI on APJ. These data support the hypothesis that APJ plays an important role in the antioxidative stress mechanism of SMI.

### 3.5. SMI Attenuated H_2_O_2_-Induced PC12 Cell Apoptosis

**Figure 5:**
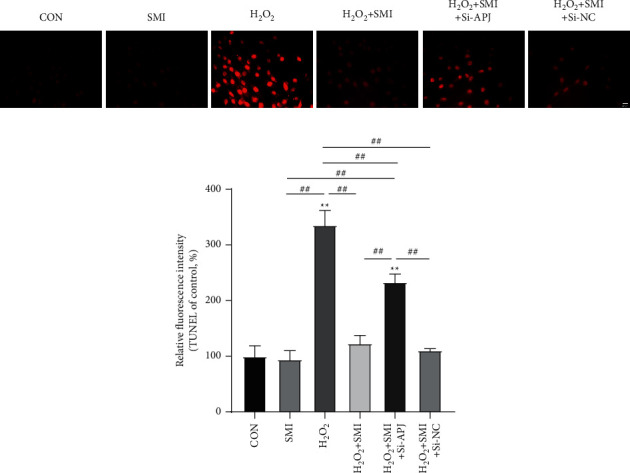
The representative images from TUNEL staining and fluorescence intensity of TUNEL. Scale bar = 20 *μ*M. ^*∗∗*^*P* < 0.01 vs control group; ^##^*P* < 0.01.

The TUNEL response is well known as apoptosis. Compared with normal PC12 cells, H_2_O_2_ stimulation significantly increased the proportion of TUNEL-positive cells and increased the cell fluorescence intensity, while SMI itself has no effect on cell apoptosis (Figure 5). Compared with the H_2_O_2_ group, SMI significantly reduced the apoptosis index, while Si-APJ partially reversed the effect of SMI. These data indicate that SMI can reduce cell apoptosis caused by H_2_O_2_, and the mechanism is related to the APJ pathway.

### 3.6. SMI Attenuated H_2_O_2_-Induced PC12 Cell Oxidative Stress

**Figure 6:**
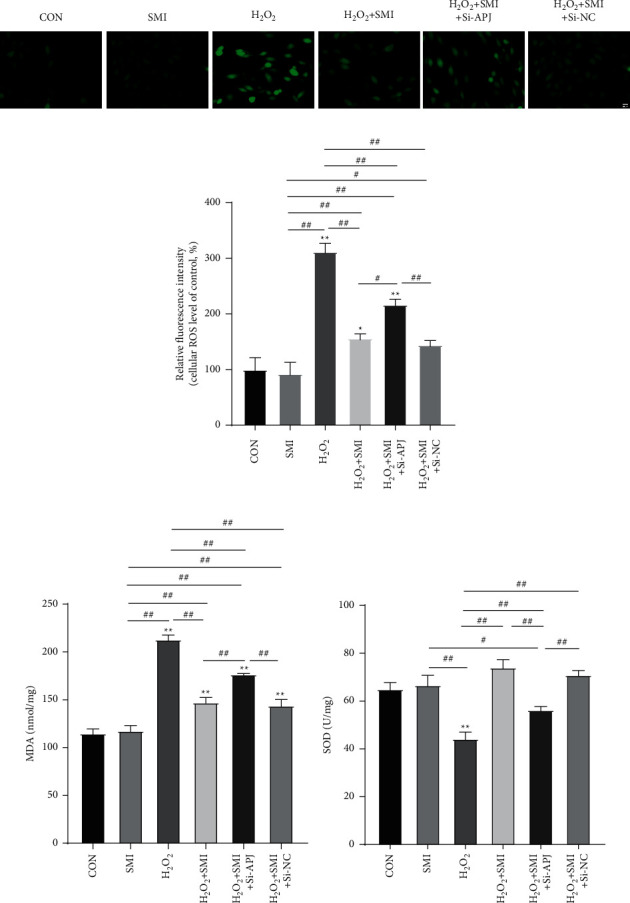
SMI attenuated H_2_O_2_-induced PC12 cells oxidative stress. (a, b) The representative images from ROS staining and fluorescence intensity of ROS. Scale bar = 20 *μ*M. (c, d) The expression levels of MDA and SOD in PC12 cells; ^*∗∗*^*P* < 0.01 vs control group; ^#^*P* < 0.05; ^##^*P* < 0.01.

To investigate the effects of SMI on H_2_O_2_-induced oxidative stress in PC12 cells, biochemical indices of oxidative stress, including SOD activity, ROS level, and MDA level, were detected. As shown in Figure 6, compared with the control group, H_2_O_2_ treatment can induce oxidative stress injury, showing that the MDA level and ROS fluorescence intensity are significantly upregulated, while SOD activity is significantly decreased. Compared with H_2_O_2_ group, SMI significantly increased SOD activity of PC12 cells, while the MDA level and ROS fluorescence intensity were significantly decreased. The effect of SMI was partially reversed by Si-APJ. The above results indicate that SMI can significantly reduce the abnormal oxidation of PC12 cells induced by H_2_O_2_ and restore the endogenous antioxidant system, while the silence of APJ weakens its antioxidant activity.

### 3.7. Effect of SMI on APJ/AMPK/GSK-3*β* Pathway

**Figure 7:**
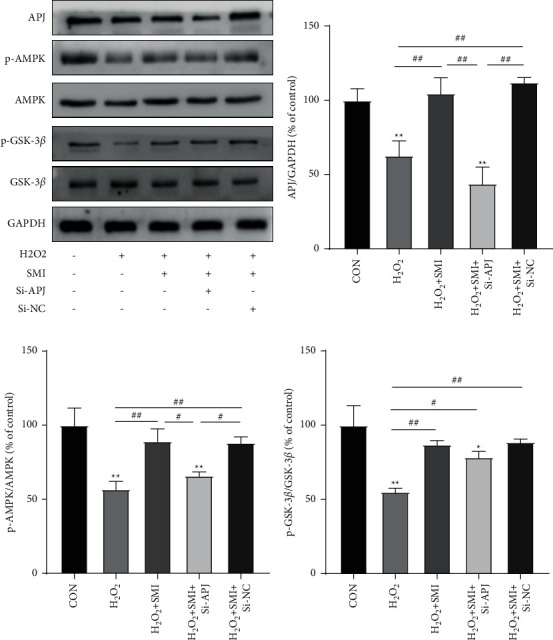
SMI alleviated H_2_O_2_ injury in PC12 cells through APJ/AMPK/GSK-3*β* signal pathway. Protein levels detected by western blot. ^*∗∗*^*P* < 0.01 VS control group; ^*∗*^*P* < 0.05VS control group; ^#^*P* < 0.05; ^##^*P* < 0.01.

To further explore the molecular mechanism downstream of APJ, the protein expression of APJ/AMPK/GSK-3*β* pathway was detected by western blot. As shown in Figure 7, H_2_O_2_ significantly reduced the protein expression levels of APJ and p-AMPK, as well as the phosphorylation level of GSK-3*β*. SMI could increase the protein expression levels of APJ, p-AMPK, and p-GSK-3*β*. When Si-APJ silenced APJ, the effects of SMI on p-AMPK and p-GSK-3*β* were significantly reduced. These results indicated that SMI could protect H_2_O_2_-damaged PC12 cells through APJ/AMPK/GSK-3*β* pathway.

## 4. Discussion

SMI is derived from the classic formula of TCM (Shengmai San) and is widely used in diseases of the cerebrovascular system, cardiovascular system, and tumor system with definite curative effects [35, 36]. A meta-analysis of 11 clinical studies showed that SMI was beneficial in improving the clinical efficacy of AIS [37].

Scientifically, oxidative stress is mainly the result of excessive accumulation of ROS [38], which plays an important role in the pathogenesis of I/R. In this study, the oxidative stress model of PC12 cells was induced by H_2_O_2_, and then SMI intervention was performed. The results showed that SMI can improve cell survival rate, alleviate oxidative stress injury, and inhibit apoptosis, suggesting that SMI has a neuroprotective effect. Early studies have shown that SMI's effect on intracellular Ca^2+^ homeostasis, especially in reducing phosphate inhibition, has a myocardial protective effect on postmyocardial infarction reperfusion [14]. It has been reported that ginsenoside Rb1 protects I/R-induced myocardial injury by regulating energy metabolism mediated by the RhoA signaling pathway [39]. Total saponins protect myocardial I/R injury through the AMPK pathway [40]. However, these studies on the mechanism of SMI on I/R injury are currently limited to the cardiovascular system, and there are few studies on the cerebrovascular system.

We used network pharmacology tools to demonstrate the molecular mechanism of the neuroprotective effect of SMI on I/R after AIS. Network pharmacology has been used to predict the pharmacological mechanisms of TCM [41, 42] and helps clarify the mechanism of action of TCM from a systematic point of view at the molecular level [43]. In this study, network pharmacology studies have shown that the 24 potential components in SMI may play a central role in regulating 362 targets that are mainly related to AIS. APJ signaling pathway is an essential pathway in the target disease-function pathway network.

At different stages of AIS, the expression of APJ will change temporarily [44]. Many transcription factors are involved in regulating the expression of APJ [45]. In the early stage of cerebral ischemia, hypoxia-inducible factor 1*α* (HIF-1*α*) and Sp1 transcription factor (Sp1) induce the upregulation of APJ expression [46, 47]. In the reperfusion phase, APJ expression is downregulated, which may be related to oxidative stress, endoplasmic reticulum, autophagy, and inflammation and the interaction between them [48, 49]. In this study, the oxidative stress induced by H_2_O_2_ downregulated the expression of APJ.

Studies have shown that AMPK is a downstream target of APJ-mediated anti-inflammatory and antioxidative stress during brain and heart ischemic injury [50, 51]. Consistent with these views, in this study, when Si-APJ was used to silence APJ, AMPK expression was also reduced. AMPK, as an energy sensor and master regulator of metabolism, plays a key role in regulating cell survival in vivo and in vitro [52]. Activating AMPK to inhibit neuronal apoptosis is considered to be a treatment strategy for neurological diseases [53]. Glycogen synthase kinase-3 (GSK-3) is a serine/threonine protein kinase, composed of two subtypes GSK-3*α* and GSK-3*β*, and is involved in a variety of cellular processes, including apoptosis, oxidative stress, cell proliferation, and glycogen metabolism [54]. AMPK inhibits GSK-3*β* activity by phosphorylation at Ser9 [44]. Recent evidence suggests that GSK-3*β* promotes cell death and that inhibition of GSK-3*β* is related to the survival mechanisms against various stresses associated with oxidative stress [55]. Our study detected the change of APJ expression in H_2_O_2_-induced cellular oxidative stress model for the first time, indicating that the neuroprotective effect of SMI is partly achieved through the APJ/AMPK/GSK-3*β* pathway.

## 5. Conclusions

In short, network pharmacology analysis shows that SMI has the characteristics of multiple components, multiple targets, and multiple pathways. Further cell experiments confirmed that it can reduce H_2_O_2_-induced oxidative stress and improve cell survival, and these mechanisms may involve activation of APJ/AMPK/GSK-3*β* signaling pathway. However, further work is needed to validate other signaling pathways and clarify their relationships.

## Data Availability

The data used to find the results of this study are provided in Supplementary Information files.

## Supplementary Materials

Supplementary Materials Table S1: drug targets information. Table S2: disease targets information. Table S3: intersection of drug and disease targets.
