# Supplementary material for: The Neuroprotective Effect of Shenmai Injection on Oxidative Stress Injury in PC12 Cells Based on Network Pharmacology
Source: Evid Based Complement Alternat Med. 2022 May 27;2022:6969740. doi: 10.1155/2022/6969740 (PMC9166949; doi:10.1155/2022/6969740)
Supplement: Supplementary Materials — Table S1: drug targets information. Table S2: disease targets information. Table S3: intersection of drug and disease targets. [file 6969740.f1.zip › 6969740.f1/Supplementary Table S1.pdf]

| Red ginseng | | Ophiopogonis Radix | |
|---|---|---|---|
| component | target | component | target |
| ginsenoside Rh4 | STAT3 | methyl ophiopogonanone b | ABCG2 |
| HEXACOSANE | SHBG | methyl ophiopogonanone b | ESR1 |
| HEXACOSANE | SPHK2 | methyl ophiopogonanone b | ESR2 |
| Octacosane | SHBG | methyl ophiopogonanone b | HSD17B1 |
| N tetracosane | SHBG | methyl ophiopogonanone b | CYP19A1 |
| Heptacosane | SHBG | methyl ophiopogonanone b | ADORA1 |
| Heptacosane | SPHK2 | methyl ophiopogonanone b | ADORA3 |
| beta-sitosterol | AR | methyl ophiopogonanone b | CA12 |
| beta-sitosterol | HMGCR | methyl ophiopogonanone b | CA7 |
| beta-sitosterol | CYP51A1 | methyl ophiopogonanone b | CYP1B1 |
| beta-sitosterol | NPC1L1 | methyl ophiopogonanone b | SRC |
| beta-sitosterol | NR1H3 | methyl ophiopogonanone b | GRM2 |
| beta-sitosterol | CYP19A1 | methyl ophiopogonanone b | CA4 |
| beta-sitosterol | CYP17A1 | methyl ophiopogonanone b | MAOB |
| beta-sitosterol | RORC | methyl ophiopogonanone b | ABCC1 |
| beta-sitosterol | ESR1 | methyl ophiopogonanone b | PTGS1 |
| beta-sitosterol | ESR2 | methyl ophiopogonanone b | SHBG |
| beta-sitosterol | SREBF2 | methyl ophiopogonanone b | KIT |
| beta-sitosterol | SHBG | methyl ophiopogonanone b | MET |
| beta-sitosterol | SLC6A2 | methyl ophiopogonanone b | CTSB |
| beta-sitosterol | CYP2C19 | methyl ophiopogonanone b | KDR |
| beta-sitosterol | RORA | methyl ophiopogonanone b | CDK4 |
| beta-sitosterol | PTPN1 | methyl ophiopogonanone b | TAS2R31 |
| beta-sitosterol | BCHE | methyl ophiopogonanone b | CTSL |
| beta-sitosterol | SERPINA6 | methyl ophiopogonanone b | CBR1 |
| beta-sitosterol | SLC6A4 | methyl ophiopogonanone b | BACE1 |
| beta-sitosterol | CHRM2 | methyl ophiopogonanone b | MMP13 |
| beta-sitosterol | VDR | methyl ophiopogonanone b | THRA |
| beta-sitosterol | ACHE | methyl ophiopogonanone b | THRB |
| beta-sitosterol | G6PD | methyl ophiopogonanone b | CDK2 |
| beta-sitosterol | NR1H2 | methyl ophiopogonanone b | ASF1A |
| beta-sitosterol | GLRA1 | methyl ophiopogonanone b | DNM1 |
| beta-sitosterol | CES2 | methyl ophiopogonanone b | CES1 |
| beta-sitosterol | PTGER1 | methyl ophiopogonanone b | CES2 |
| beta-sitosterol | PTGER2 | methyl ophiopogonanone b | FLT3 |
| beta-sitosterol | HSD11B1 | methyl ophiopogonanone b | PTGS2 |
| beta-sitosterol | PTGES | methyl ophiopogonanone b | SIRT2 |
| beta-sitosterol | CDC25A | methyl ophiopogonanone b | FGFR1 |
| beta-sitosterol | PPARA | methyl ophiopogonanone b | CHRNA7 |
| beta-sitosterol | PPARD | methyl ophiopogonanone b | RAF1 |
| beta-sitosterol | DHCR7 | methyl ophiopogonanone b | BRAF |
| beta-sitosterol | SQLE | methyl ophiopogonanone b | MMP12 |
| beta-sitosterol | PTPN6 | methyl ophiopogonanone b | EPHB4 |
| beta-sitosterol | NR1I3 | methyl ophiopogonanone b | FOS |
| beta-sitosterol | FDFT1 | methyl ophiopogonanone b | ABL1 |

| | | | |
|---|---|---|---|
| beta-sitosterol | SIGMAR1 | methyl ophiopogonanone b | EPHA2 |
| beta-sitosterol | NOS2 | methyl ophiopogonanone b | LCK |
| beta-sitosterol | NR3C1 | methyl ophiopogonanone b | EPHB2 |
| beta-sitosterol | PPARG | methyl ophiopogonanone b | EPHA5 |
| beta-sitosterol | CDC25B | methyl ophiopogonanone b | EPHA4 |
| beta-sitosterol | UGT2B7 | methyl ophiopogonanone b | EPHA8 |
| beta-sitosterol | HSD11B2 | methyl ophiopogonanone b | MKNK2 |
| beta-sitosterol | POLB | methyl ophiopogonanone b | EPHA7 |
| N nonacosane | SHBG | methyl ophiopogonanone b | EPHB3 |
| Ginsenoside Rh1 | STAT3 | methyl ophiopogonanone b | EPHA3 |
| 20(R)-Protopanaxatri | PTPN1 | methyl ophiopogonanone b | EPHB1 |
| 20(R)-Protopanaxatri | CYP2C19 | methyl ophiopogonanone b | EPHA1 |
| 20(R)-Protopanaxatri | CHRM2 | methyl ophiopogonanone b | HSD17B2 |
| 20(R)-Protopanaxatri | SLC6A2 | methyl ophiopogonanone b | RPS6KB1 |
| 20(R)-Protopanaxatri | SLC6A4 | methyl ophiopogonanone b | AURKA |
| 20(R)-Protopanaxatri | AR | methyl ophiopogonanone b | AURKB |
| 20(R)-Protopanaxatri | ACHE | methyl ophiopogonanone b | F3 |
| 20(R)-Protopanaxatri | HSD11B1 | methyl ophiopogonanone b | PTPN1 |
| 20(R)-Protopanaxatri | ESR1 | methyl ophiopogonanone b | SERPINE1 |
| 20(R)-Protopanaxatri | CYP19A1 | methyl ophiopogonanone b | HSP90AB1 |
| 20(R)-Protopanaxatri | ATP12A | methyl ophiopogonanone b | CHEK1 |
| 20(R)-Protopanaxatri | NR1H3 | methyl ophiopogonanone b | PDK1 |
| 20(R)-Protopanaxatri | HMGCR | methyl ophiopogonanone b | WEE1 |
| 20(R)-Protopanaxatri | CYP51A1 | methyl ophiopogonanone b | BCL2L1 |
| 20(R)-Protopanaxatri | NPC1L1 | methyl ophiopogonanone b | POLB |
| 20(R)-Protopanaxatri | RORC | methyl ophiopogonanone b | MELK |
| 20(R)-Protopanaxatri | SREBF2 | methyl ophiopogonanone b | EIF2AK2 |
| 20(R)-Protopanaxatri | CYP17A1 | methyl ophiopogonanone b | ALOX12 |
| 20(R)-Protopanaxatri | PTGS2 | methyl ophiopogonanone b | ANPEP |
| 20(R)-Protopanaxatri | PDE10A | methyl ophiopogonanone b | CDK9 |
| 20(R)-Protopanaxatri | UGT2B7 | methyl ophiopogonanone b | SYK |
| 20(R)-Protopanaxatri | NR1I3 | methyl ophiopogonanone b | CCNA2 |
| 20(R)-Protopanaxatri | PTGES | methyl ophiopogonanone b | CDK5 |
| 20(R)-Protopanaxatri | PIK3CB | methyl ophiopogonanone b | CLK4 |
| 20(R)-Protopanaxatri | ALK | methyl ophiopogonanone b | CLK2 |
| 20(R)-Protopanaxatri | PDE2A | methyl ophiopogonanone b | CLK3 |
| 20(R)-Protopanaxatri | PDE4B | methyl ophiopogonanone b | DYRK2 |
| 20(R)-Protopanaxatri | PIK3CD | methyl ophiopogonanone b | DYRK3 |
| 20(R)-Protopanaxatri | PIK3CG | methyl ophiopogonanone b | AKR1B1 |
| 20(R)-Protopanaxatri | PIK3CA | methyl ophiopogonanone b | AKT1 |
| 20(R)-Protopanaxatri | TTL | methyl ophiopogonanone b | PLA2G5 |
| 20(R)-Protopanaxatri | MAPK8 | methyl ophiopogonanone b | PLA2G10 |
| 20(R)-Protopanaxatri | SMO | methyl ophiopogonanone b | STAT6 |
| 20(R)-Protopanaxatri | DRD1 | methyl ophiopogonanone b | HSP90AA1 |
| 20(R)-Protopanaxatri | DRD2 | methyl ophiopogonanone b | BMP1 |
| 20(R)-Protopanaxatri | DRD3 | methyl ophiopogonanone b | ALOX15 |
| 20(R)-Protopanaxatri | MTOR | methyl ophiopogonanone b | PLA2G1B |

| | | |
|---|---|---|
| 20(R)-ProtopanaxatriBCHE | methyl ophiopogonanone b | LTB4R |
| 20(R)-ProtopanaxatriPSEN2 | methyl ophiopogonanone b | GRM5 |
| 20(R)-ProtopanaxatriCCNC CDK8 | methyl ophiopogonanone b | KLK1 |
| 20(R)-ProtopanaxatriCDK8 | methyl ophiopogonanone b | KLK2 |
| 20(R)-ProtopanaxatriPPP1CC | methyl ophiopogonanone b | TRPM8 |
| 20(R)-ProtopanaxatriPPP2CA | methyl ophiopogonanone b | RXRA |
| 20(R)-ProtopanaxatriVDR | methyl ophiopogonanone b | HNF4A |
| 20(R)-ProtopanaxatriAVPR1A | methyl ophiopogonanone b | SLC29A1 |
| 20(R)-ProtopanaxatriCAPN2 | methyl ophiopogonanone b | JUN |
| 20(R)-ProtopanaxatriINSR | methyl ophiopogonanone b | CCNT1 |
| 20(R)-ProtopanaxatriJAK1 | methyl ophiopogonanone b | CDK2 |
| 20(R)-ProtopanaxatriJAK2 | Ophiopogonin B | STAT3 |
| 20(R)-ProtopanaxatriF2R | Ophiopogonin D | IL2 |
| 20(R)-ProtopanaxatriCRHR1 | Ophiopogonin D | STAT3 |
| 20(R)-ProtopanaxatriCYP2C9 | Stigmasterol | ESR1 |
| 20(R)-ProtopanaxatriCOL4A3BP | Stigmasterol | SLC6A2 |
| 20(R)-ProtopanaxatriCYP3A4 | Stigmasterol | CYP2C19 |
| 20(R)-ProtopanaxatriGPR55 | Stigmasterol | CYP19A1 |
| 20(R)-ProtopanaxatriGPR18 | Stigmasterol | PPARA |
| 20(R)-ProtopanaxatriITGAL | Stigmasterol | CNR2 |
| 20(R)-ProtopanaxatriPYGL | Stigmasterol | SLC6A4 |
| 20(R)-ProtopanaxatriSTAT3 | Stigmasterol | CHRM2 |
| 20(R)-ProtopanaxatriPTGS1 | Stigmasterol | ACHE |
| 20(R)-ProtopanaxatriHSP90AA1 | Stigmasterol | PTPN1 |
| 20(R)-ProtopanaxatriSCN9A | Stigmasterol | AR |
| 20(R)-ProtopanaxatriCCNT1 | Stigmasterol | SIGMAR1 |
| 20(R)-ProtopanaxatriPCSK7 | Stigmasterol | SREBF2 |
| 20(R)-ProtopanaxatriOPRM1 | Stigmasterol | NPC1L1 |
| 20(R)-ProtopanaxatriOPRD1 | Stigmasterol | ESR2 |
| 20(R)-ProtopanaxatriCYP2D6 | Stigmasterol | CTSD |
| 20(R)-ProtopanaxatriACACB | Stigmasterol | SHBG |
| 20(R)-ProtopanaxatriMAPK14 | Stigmasterol | HMGCR |
| 20(R)-ProtopanaxatriALPL | Stigmasterol | CYP51A1 |
| 20(R)-ProtopanaxatriPDE5A | Stigmasterol | CXCR3 |
| 20(R)-ProtopanaxatriADORA1 | Stigmasterol | SLC18A2 |
| 20(R)-ProtopanaxatriADORA2A | Stigmasterol | SLC6A9 |
| 20(R)-ProtopanaxatriADORA2B | Stigmasterol | SRD5A1 |
| 20(R)-ProtopanaxatriCALCRL | Stigmasterol | SRD5A2 |
| 20(R)-ProtopanaxatriCCR2 | Stigmasterol | SERPINA6 |
| 20(R)-ProtopanaxatriHCRTR2 | Stigmasterol | NR1I2 |
| 20(R)-ProtopanaxatriHCRTR1 | Stigmasterol | HSD17B3 |
| 20(R)-ProtopanaxatriPGC | Stigmasterol | FABP1 |
| 20(R)-ProtopanaxatriCTSD | ophiopogonanone A | CA12 |
| 20(R)-ProtopanaxatriREN | ophiopogonanone A | MET |
| 20(R)-ProtopanaxatriCTSE | ophiopogonanone A | KDR |
| 20(R)-ProtopanaxatriPGA5 | ophiopogonanone A | CYP1B1 |
| 20(R)-ProtopanaxatriPSENEN | ophiopogonanone A | CA7 |

| | | | |
|---|---|---|---|
| 20(R)-Protopanaxatri | NCSTN | ophiopogonanone A | ABCG2 |
| 20(R)-Protopanaxatri | APH1A | ophiopogonanone A | SRC |
| 20(R)-Protopanaxatri | PSEN1 | ophiopogonanone A | ESR1 |
| 20(R)-Protopanaxatri | APH1B | ophiopogonanone A | ESR2 |
| Ginsenoside RG1 | STAT3 | ophiopogonanone A | CYP19A1 |
| 20(S)-Ginsenoside Rh | STAT3 | ophiopogonanone A | HSD17B1 |
| | | ophiopogonanone A | KIT |
| | | ophiopogonanone A | PARP1 |
| | | ophiopogonanone A | CA4 |
| | | ophiopogonanone A | RAF1 |
| | | ophiopogonanone A | BRAF |
| | | ophiopogonanone A | ADORA1 |
| | | ophiopogonanone A | ADORA3 |
| | | ophiopogonanone A | CHEK1 |
| | | ophiopogonanone A | HSP90AA1 |
| | | ophiopogonanone A | ABCC1 |
| | | ophiopogonanone A | MTOR |
| | | ophiopogonanone A | PIK3CA |
| | | ophiopogonanone A | FGFR1 |
| | | ophiopogonanone A | LNPEP |
| | | ophiopogonanone A | CHRNA7 |
| | | ophiopogonanone A | MAOB |
| | | ophiopogonanone A | MMP8 |
| | | ophiopogonanone A | PIK3CD |
| | | ophiopogonanone A | PIK3CB |
| | | ophiopogonanone A | PIK3CG |
| | | ophiopogonanone A | F3 |
| | | ophiopogonanone A | LTB4R |
| | | ophiopogonanone A | GCGR |
| | | ophiopogonanone A | SLC9A1 |
| | | ophiopogonanone A | RET |
| | | ophiopogonanone A | PDK1 |
| | | ophiopogonanone A | HSD17B2 |
| | | ophiopogonanone A | ALOX15 |
| | | ophiopogonanone A | MMP13 |
| | | ophiopogonanone A | CA2 |
| | | ophiopogonanone A | CA1 |
| | | ophiopogonanone A | CA14 |
| | | ophiopogonanone A | CDK5R1 |
| | | ophiopogonanone A | PIM1 |
| | | ophiopogonanone A | CLK1 |
| | | ophiopogonanone A | CLK3 |
| | | ophiopogonanone A | DYRK2 |
| | | ophiopogonanone A | DBF4 |
| | | ophiopogonanone A | EIF2AK2 |
| | | ophiopogonanone A | CCNE2 |
| | | ophiopogonanone A | ENPP2 |

| | |
|---|---|
| ophiopogonanone A | YWHAG |
| ophiopogonanone A | SYK |
| ophiopogonanone A | WEE1 |
| ophiopogonanone A | CDK2 |
| ophiopogonanone A | CDK4 |
| ophiopogonanone A | MAP3K8 |
| ophiopogonanone A | ANPEP |
| ophiopogonanone A | ALOX12 |
| ophiopogonanone A | AURKA |
| ophiopogonanone A | SHBG |
| ophiopogonanone A | DNM1 |
| ophiopogonanone A | EZR |
| ophiopogonanone A | ATP4B |
| ophiopogonanone A | RPS6KA3 |
| ophiopogonanone A | ADORA2A |
| ophiopogonanone A | MMP12 |
| ophiopogonanone A | ABL1 |
| ophiopogonanone A | PRKDC |
| ophiopogonanone A | HCK |
| ophiopogonanone A | EPHB4 |
| ophiopogonanone A | ERN1 |
| ophiopogonanone A | FLT3 |
| ophiopogonanone A | IKBKB |
| ophiopogonanone A | JAK3 |
| ophiopogonanone A | PDPK1 |
| ophiopogonanone A | LCK |
| ophiopogonanone A | GSK3B |
| ophiopogonanone A | JAK2 |
| ophiopogonanone A | ALK |
| ophiopogonanone A | VCP |
| ophiopogonanone A | CCNE1 |
| ophiopogonanone A | AURKB |
| ophiopogonanone A | CCNE1 |
| ophiopogonanone A | BCL2L1 |
| ophiopogonanone A | MMP25 |
| ophiopogonanone A | CDK9 |
| ophiopogonanone A | MMP16 |
| ophiopogonanone A | CCNA2 |
| ophiopogonanone A | CDK1 |
| ophiopogonanone A | ADCY5 |
| ophiopogonanone A | ALPG |
| ophiopogonanone A | BMP1 |
| ophiopogonanone A | CDK5 |
| ophiopogonanone A | MMP7 |
| ophiopogonanone A | CLK4 |
| ophiopogonanone A | CLK2 |
| ophiopogonanone A | DYRK3 |

| | |
|---|---|
| ophiopogonanone A | ADAM10 |
| ophiopogonanone A | CDK5 |
| ophiopogonanone A | CDC7 |
| ophiopogonanone A | CDK2 |
| ophiopogonanone A | ATP4A |
| ophiopogonanone A | CDK2 |
| ophiopogonanone A | CDK3 |
| ophiopogonanone A | CCNT1 |
| ophiopogonanone A | CCNE1 |
| ophiopogonanone C | KIT |
| ophiopogonanone C | HSP90AB1 |
| ophiopogonanone C | CBR1 |
| ophiopogonanone C | HSP90AA1 |
| ophiopogonanone C | HSP90B1 |
| ophiopogonanone C | FGFR1 |
| ophiopogonanone C | CA4 |
| ophiopogonanone C | GCGR |
| ophiopogonanone C | SIRT1 |
| ophiopogonanone C | TAS2R31 |
| ophiopogonanone C | ESR1 |
| ophiopogonanone C | ESR2 |
| ophiopogonanone C | CYP19A1 |
| ophiopogonanone C | LTB4R |
| ophiopogonanone C | CA12 |
| ophiopogonanone C | CTSB |
| ophiopogonanone C | PDE10A |
| ophiopogonanone C | EGLN1 |
| ophiopogonanone C | DNASE1L3 |
| ophiopogonanone C | CYP1B1 |
| ophiopogonanone C | GRM2 |
| ophiopogonanone C | MET |
| ophiopogonanone C | PTGS2 |
| ophiopogonanone C | ADORA1 |
| ophiopogonanone C | NOX4 |
| ophiopogonanone C | SHBG |
| ophiopogonanone C | CFTR |
| ophiopogonanone C | OPRD1 |
| ophiopogonanone C | ALOX15 |
| ophiopogonanone C | PDE5A |
| ophiopogonanone C | ABCG2 |
| ophiopogonanone C | CA7 |
| ophiopogonanone C | CDK1 |
| ophiopogonanone C | NOS2 |
| ophiopogonanone C | IKBKB |
| ophiopogonanone C | EDNRA |
| ophiopogonanone C | ALOX12 |
| ophiopogonanone C | AKT1 |

| | |
|---|---|
| ophiopogonanone C | CTSL |
| ophiopogonanone C | BCL2L1 |
| ophiopogonanone C | TNNC1 |
| ophiopogonanone C | ALPL |
| ophiopogonanone C | SRC |
| ophiopogonanone C | CDK4 |
| ophiopogonanone C | BACE1 |
| ophiopogonanone C | PIK3CG |
| ophiopogonanone C | TNNT2 |
| ophiopogonanone C | TNNI3 |
| ophiopogonanone e | TAS2R31 |
| ophiopogonanone e | ABCG2 |
| ophiopogonanone e | MET |
| ophiopogonanone e | KDR |
| ophiopogonanone e | SRC |
| ophiopogonanone e | KIT |
| ophiopogonanone e | FGFR1 |
| ophiopogonanone e | CYP1B1 |
| ophiopogonanone e | ESR1 |
| ophiopogonanone e | ESR2 |
| ophiopogonanone e | CA7 |
| ophiopogonanone e | CA12 |
| ophiopogonanone e | CA4 |
| ophiopogonanone e | ADORA1 |
| ophiopogonanone e | ADORA3 |
| ophiopogonanone e | CDK2 |
| ophiopogonanone e | CDK4 |
| ophiopogonanone e | HSP90AB1 |
| ophiopogonanone e | GUSB |
| ophiopogonanone e | PTGS1 |
| ophiopogonanone e | ALOX12 |
| ophiopogonanone e | HSD17B1 |
| ophiopogonanone e | MMP13 |
| ophiopogonanone e | EIF2AK2 |
| ophiopogonanone e | CDK1 |
| ophiopogonanone e | KCNA3 |
| ophiopogonanone e | CYP19A1 |
| ophiopogonanone e | CHRNA7 |
| ophiopogonanone e | EPHB4 |
| ophiopogonanone e | BRAF |
| ophiopogonanone e | ALOX15 |
| ophiopogonanone e | PARP1 |
| ophiopogonanone e | SGK1 |
| ophiopogonanone e | MAOB |
| ophiopogonanone e | AURKB |
| ophiopogonanone e | RPS6KB1 |
| ophiopogonanone e | AURKA |

| | |
|---|---|
| ophiopogonanone e | DUSP3 |
| ophiopogonanone e | MMP12 |
| ophiopogonanone e | CA3 |
| ophiopogonanone e | BACE1 |
| ophiopogonanone e | FLT3 |
| ophiopogonanone e | MKNK2 |
| ophiopogonanone e | CA2 |
| ophiopogonanone e | CA1 |
| ophiopogonanone e | CDK2 |
| ophiopogonanone e | LTB4R |
| ophiopogonanone e | SPHK2 |
| ophiopogonanone e | SPHK1 |
| ophiopogonanone e | ALOX5 |
| ophiopogonanone e | SFRP1 |
| ophiopogonanone e | GCGR |
| ophiopogonanone e | ASF1A |
| ophiopogonanone e | AKT1 |
| ophiopogonanone e | CA6 |
| ophiopogonanone e | CA13 |
| ophiopogonanone e | CA5B |
| ophiopogonanone e | CA5A |
| ophiopogonanone e | MMP8 |
| ophiopogonanone e | CHEK1 |
| ophiopogonanone e | WEE1 |
| ophiopogonanone e | TGFBR1 |
| ophiopogonanone e | ACHE |
| ophiopogonanone e | HMGCR |
| ophiopogonanone e | FOS |
| ophiopogonanone e | KLK1 |
| ophiopogonanone e | KLK2 |
| ophiopogonanone e | ABCC1 |
| ophiopogonanone e | BMP1 |
| ophiopogonanone e | TNF |
| ophiopogonanone e | CTSB |
| ophiopogonanone e | HPGDS |
| ophiopogonanone e | CCNA1 |
| ophiopogonanone e | JUN |
| ophiopogonanone e | CCNA2 |
| uridine | CDA |
| uridine | ADA |
| uridine | PYGM |
| uridine | TK1 |
| orchinol | ALOX5 |
| orchinol | HSD17B3 |
| orchinol | CYP11B1 |
| orchinol | CYP11B2 |
| orchinol | CYP17A1 |

| | |
|---|---|
| orchinol | CDK5R1 |
| orchinol | CCNE2 |
| orchinol | CCNB3 |
| orchinol | CDK2 |
| orchinol | GSK3B |
| orchinol | GSK3A |
| orchinol | CDK5 |
| orchinol | CFTR |
| orchinol | DCTPP1 |
| orchinol | QDPR |
| orchinol | CHRNA3 |
| orchinol | CHRNA7 |
| orchinol | TRPV1 |
| orchinol | HTT |
| orchinol | SIGMAR1 |
| orchinol | MMP9 |
| orchinol | MMP1 |
| orchinol | MMP2 |
| orchinol | MAOB |
| orchinol | CCND3 |
| orchinol | HDAC6 |
| orchinol | HDAC1 |
| orchinol | BRAF |
| orchinol | ABL1 |
| orchinol | EGFR |
| orchinol | SRC |
| orchinol | PRKCA |
| orchinol | CDK1 |
| orchinol | MET |
| orchinol | NQO2 |
| orchinol | DYRK1A |
| orchinol | LNPEP |
| orchinol | COMT |
| orchinol | CA1 |
| orchinol | CA12 |
| orchinol | CA14 |
| orchinol | MAP2K1 |
| orchinol | CA9 |
| orchinol | CA4 |
| orchinol | CA13 |
| orchinol | CA5B |
| orchinol | CA5A |
| orchinol | ADORA1 |
| orchinol | ERBB2 |
| orchinol | CA2 |
| orchinol | MAPKAPK2 |
| orchinol | HSD17B2 |

| | |
|---|---|
| orchinol | MAOA |
| orchinol | MIF |
| orchinol | AKT1 |
| orchinol | DAO |
| orchinol | ALPG |
| orchinol | PLAA |
| orchinol | CNR2 |
| orchinol | GRM5 |
| orchinol | OPRD1 |
| orchinol | MKNK1 |
| orchinol | RET |
| orchinol | KDR |
| orchinol | RAF1 |
| orchinol | JAK2 |
| orchinol | PARP1 |
| orchinol | TNKS2 |
| orchinol | TNKS |
| orchinol | MME |
| orchinol | ESR1 |
| orchinol | ESR2 |
| orchinol | AR |
| orchinol | CDK4 |
| orchinol | PIM1 |
| orchinol | GABRB3 |
| orchinol | PLA2G2A |
| orchinol | FGFR1 |
| orchinol | TGFBR1 |
| orchinol | RPS6KA3 |
| orchinol | OPRK1 |
| orchinol | MGLL |
| orchinol | CDC25B |
| orchinol | LCK |
| orchinol | TUBB3 |
| orchinol | PSEN2 |
| orchinol | CDC25A |
| orchinol | RPS6KA2 |
| orchinol | QPCT |
| orchinol | MTOR |
| orchinol | PIK3CA |
| orchinol | JAK3 |
| orchinol | ALK |
| orchinol | KIF11 |
| orchinol | CHEK1 |
| orchinol | ADORA2A |
| orchinol | ADORA2B |
| orchinol | ADORA3 |
| orchinol | PGR |

| | |
|---|---|
| orchinol | MAPK14 |
| orchinol | CDK5 |
| orchinol | CDK2 |
| orchinol | CDK1 |
| orchinol | CCNA1 |
| orchinol | CHRNB4 |
| orchinol | CCND1 |
| orchinol | GABRG2 |
| orchinol | PSENEN |
| orchinol | CCNE1 |
| orchinol | CCNB1 |
| orchinol | CCNA2 |
| orchinol | CDK4 |
| orchinol | GABRA5 |
| orchinol | NCSTN |
| orchinol | CCNB2 |
| orchinol | CCND2 |
| orchinol | APH1A |
| orchinol | PSEN1 |
| orchinol | APH1B |
| ophiopogon A | STAT3 |
| ophiopogonone A | HSP90AB1 |
| ophiopogonone A | NQO1 |
| ophiopogonone A | HSP90AA1 |
| ophiopogonone A | CNR1 |
| ophiopogonone A | HSP90B1 |
| ophiopogonone A | GCGR |
| ophiopogonone A | CDK1 |
| ophiopogonone A | GPR55 |
| ophiopogonone A | AKR1B1 |
| ophiopogonone A | ALPL |
| ophiopogonone A | CHEK1 |
| ophiopogonone A | GRIN2B |
| ophiopogonone A | CDC7 |
| ophiopogonone A | CDC25B |
| ophiopogonone A | CDK5R1 |
| ophiopogonone A | DNASE1L3 |
| ophiopogonone A | OPRM1 |
| ophiopogonone A | PIK3CG |
| ophiopogonone A | TNNC1 |
| ophiopogonone A | CDK5 |
| ophiopogonone A | TNNT2 |
| ophiopogonone A | TNNI3 |
| ophiopogonone B | NQO1 |
| ophiopogonone B | CNR1 |
| ophiopogonone B | HSP90AB1 |
| ophiopogonone B | GPR55 |

| | |
|---|---|
| ophiopogonone B | HSP90AA1 |
| ophiopogonone B | GCGR |
| ophiopogonone B | CYP11B1 |
| ophiopogonone B | CYP11B2 |
| ophiopogonone B | ALPL |
| ophiopogonone B | ABCB1 |
| ophiopogonone B | AKR1B1 |
| ophiopogonone B | CNR2 |
| ophiopogonone B | PCSK7 |
| ophiopogonone B | CYP2C9 |
| ophiopogonone B | HSP90B1 |
| ophiopogonone B | SHBG |
| ophiopogonone B | CYP19A1 |
| ophiopogonone B | PDE10A |
| ophiopogonone B | CBR1 |
| ophiopogonone B | CFTR |
| ophiopogonone B | GPR84 |
| ophiopogonone B | PDK1 |
| ophiopogonone B | AR |
| ophiopogonone B | GRIN2B |
| ophiopogonone B | CHEK1 |
| ophiopogonone B | MAOA |
| ophiopogonone B | CLK1 |
| ophiopogonone B | HSD17B3 |
| ophiopogonone B | CCNB3 |
| ophiopogonone B | CDK1 |
| ophiopogonone B | CCNB1 |
| ophiopogonone B | CCNB2 |
| N-Trans-Feruloyltyramine | MMP9 |
| N-Trans-Feruloyltyramine | MMP2 |
| N-Trans-Feruloyltyramine | MMP1 |
| N-Trans-Feruloyltyramine | EGFR |
| N-Trans-Feruloyltyramine | CNR2 |
| N-Trans-Feruloyltyramine | MAOB |
| N-Trans-Feruloyltyramine | TYR |
| N-Trans-Feruloyltyramine | BRAF |
| N-Trans-Feruloyltyramine | CDK1 |
| N-Trans-Feruloyltyramine | PTGS2 |
| N-Trans-Feruloyltyramine | ALOX5 |
| N-Trans-Feruloyltyramine | SYK |
| N-Trans-Feruloyltyramine | CHEK1 |
| N-Trans-Feruloyltyramine | WEE1 |
| N-Trans-Feruloyltyramine | CTSL |
| N-Trans-Feruloyltyramine | MTNR1A |
| N-Trans-Feruloyltyramine | HSP90AA1 |
| N-Trans-Feruloyltyramine | BCHE |
| N-Trans-Feruloyltyramine | ACHE |

| | |
|---|---|
| N-Trans-Feruloyltyramine | GLI2 |
| N-Trans-Feruloyltyramine | ABL1 |
| N-Trans-Feruloyltyramine | CDK5R1 |
| N-Trans-Feruloyltyramine | ALDH2 |
| N-Trans-Feruloyltyramine | MTOR |
| N-Trans-Feruloyltyramine | PDK1 |
| N-Trans-Feruloyltyramine | DNM1 |
| N-Trans-Feruloyltyramine | ESRRA |
| N-Trans-Feruloyltyramine | ESRRB |
| N-Trans-Feruloyltyramine | THRA |
| N-Trans-Feruloyltyramine | THRB |
| N-Trans-Feruloyltyramine | PRKCZ |
| N-Trans-Feruloyltyramine | RPS6KB1 |
| N-Trans-Feruloyltyramine | CHEK2 |
| N-Trans-Feruloyltyramine | BMP1 |
| N-Trans-Feruloyltyramine | MMP3 |
| N-Trans-Feruloyltyramine | DUSP3 |
| N-Trans-Feruloyltyramine | MMP8 |
| N-Trans-Feruloyltyramine | CA7 |
| N-Trans-Feruloyltyramine | CA6 |
| N-Trans-Feruloyltyramine | CA12 |
| N-Trans-Feruloyltyramine | CA14 |
| N-Trans-Feruloyltyramine | CA9 |
| N-Trans-Feruloyltyramine | CA5A |
| N-Trans-Feruloyltyramine | ROCK2 |
| N-Trans-Feruloyltyramine | HPGDS |
| N-Trans-Feruloyltyramine | DRD2 |
| N-Trans-Feruloyltyramine | DRD3 |
| N-Trans-Feruloyltyramine | HSD17B2 |
| N-Trans-Feruloyltyramine | HSD17B1 |
| N-Trans-Feruloyltyramine | ADORA1 |
| N-Trans-Feruloyltyramine | ADORA2A |
| N-Trans-Feruloyltyramine | PDE4D |
| N-Trans-Feruloyltyramine | PDE4C |
| N-Trans-Feruloyltyramine | HDAC1 |
| N-Trans-Feruloyltyramine | CDK1 |
| N-Trans-Feruloyltyramine | CCNE1 |
| N-Trans-Feruloyltyramine | CCNE1 |
| N-Trans-Feruloyltyramine | ADAM17 |
| N-Trans-Feruloyltyramine | GRK2 |
| N-Trans-Feruloyltyramine | SLC5A1 |
| N-Trans-Feruloyltyramine | MMP13 |
| N-Trans-Feruloyltyramine | MMP7 |
| N-Trans-Feruloyltyramine | TRPM8 |
| N-Trans-Feruloyltyramine | ALK |
| N-Trans-Feruloyltyramine | INSR |
| N-Trans-Feruloyltyramine | CFD |

| | |
|---|---|
| N-Trans-Feruloyltyramine | TNF |
| N-Trans-Feruloyltyramine | TOP1 |
| N-Trans-Feruloyltyramine | AGTR1 |
| N-Trans-Feruloyltyramine | ANPEP |
| N-Trans-Feruloyltyramine | MMP14 |
| N-Trans-Feruloyltyramine | PNMT |
| N-Trans-Feruloyltyramine | CDK4 |
| N-Trans-Feruloyltyramine | PTPN1 |
| N-Trans-Feruloyltyramine | TRAP1 |
| N-Trans-Feruloyltyramine | HSP90B1 |
| N-Trans-Feruloyltyramine | HTR3A |
| N-Trans-Feruloyltyramine | HSP90AB1 |
| N-Trans-Feruloyltyramine | VCP |
| N-Trans-Feruloyltyramine | AVPR1A |
| N-Trans-Feruloyltyramine | NR1H4 |
| N-Trans-Feruloyltyramine | ADORA3 |
| N-Trans-Feruloyltyramine | ADAM10 |
| N-Trans-Feruloyltyramine | TSPO |
| N-Trans-Feruloyltyramine | CA13 |
| N-Trans-Feruloyltyramine | CA5B |
| N-Trans-Feruloyltyramine | TYMS |
| N-Trans-Feruloyltyramine | MIF |
| N-Trans-Feruloyltyramine | PDE7A |
| N-Trans-Feruloyltyramine | EPHA2 |
| N-Trans-Feruloyltyramine | MMP16 |
| N-Trans-Feruloyltyramine | AKT2 |
| N-Trans-Feruloyltyramine | EPHB2 |
| N-Trans-Feruloyltyramine | MAP2K1 |
| N-Trans-Feruloyltyramine | EPHA5 |
| N-Trans-Feruloyltyramine | EPHA4 |
| N-Trans-Feruloyltyramine | EPHA8 |
| N-Trans-Feruloyltyramine | MMP12 |
| N-Trans-Feruloyltyramine | EPHA7 |
| N-Trans-Feruloyltyramine | EPHB3 |
| N-Trans-Feruloyltyramine | CDK5 |
| N-Trans-Feruloyltyramine | CCNB1 |
| N-Trans-Feruloyltyramine | CDK2 |
| N-Trans-Feruloyltyramine | CDK3 |
