# Supplementary material for: The Neuroprotective Effect of Shenmai Injection on Oxidative Stress Injury in PC12 Cells Based on Network Pharmacology
Source: Evid Based Complement Alternat Med. 2022 May 27;2022:6969740. doi: 10.1155/2022/6969740 (PMC9166949; doi:10.1155/2022/6969740)
Supplement: Supplementary Materials — Table S1: drug targets information. Table S2: disease targets information. Table S3: intersection of drug and disease targets. [file 6969740.f1.zip › 6969740.f1/Supplementary Table S2.pdf]

OMIM	GeneCards,10	OMIM+GeneCards
GNB1	F2	GNB1
TAL1	F5	TAL1
CPT2	NOTCH3	CPT2
AIR	NOS3	AIR
RBM15	TP53	RBM15
F5	JAK2	F5
LPIN1	CEBPA	LPIN1
DNMT3A	MT-TL1	DNMT3A
HADHA	RUNX1	HADHA
RANBP2	ACE	RANBP2
GATA2	IL6	GATA2
DBR1	MTHFR	DBR1
MME	FBN1	MME
LPP	ALOX5AP	LPP
CHIC2	CRP	CHIC2
KIT	TNF	KIT
RAP1GDS1	LDLR	RAP1GDS1
TERT	KIT	TERT
STRK1	PIK3CA	STRK1
ACSL6	PRKCH	ACSL6
IRF1	GP1BA	IRF1
NPM1	KMT2A	NPM1
DEK	TET2	DEK
MYB	KRAS	MYB
ALL2	HMBS	ALL2
NOS3	IL1B	NOS3
BLACE	ENG	BLACE
STAR	DNMT3A	STAR
NBN	APOE	NBN
BAALC	INSL6	BAALC
JAK2	TERT	JAK2
LALL	GATA2	LALL
PAX5	IL10	PAX5
TAL2	ACSL4	TAL2
ALAD	STAT3	ALAD
SET	NSD1	SET
NUP214	MT-ND1	NUP214
AF10	PLAT	AF10
ALL1	SERPINE1	ALL1
LMO1	MIR126	LMO1
LMO2	COL4A1	LMO2
F2	IDH1	F2
UNC93B1	MMP9	UNC93B1
NUMA1	MIR155	NUMA1
PICALM	ACTB	PICALM
ZBTB16	NPPB	ZBTB16

HMBS	CDKN2A	HMBS
ETV6	ADAMTS13	ETV6
KRAS	GATA1	KRAS
TBK1	MT-ND5	TBK1
ALDH2	SELP	ALDH2
FLT3	VEGFA	FLT3
ALOX5AP	MYH11	ALOX5AP
SNORA31	NPPA	SNORA31
TRAF3	IDH2	TRAF3
PML	AGTR1	PML
MEFV	SERPINC1	MEFV
CREBBP	PICALM	CREBBP
MYH11	ALB	MYH11
CBFB	THBD	CBFB
GP1BA	ABL1	GP1BA
RARA	PML	RARA
STAT5B	PTEN	STAT5B
STAT3	CST3	STAT3
ACE	ADA2	ACE
SH3GL1	TLR4	SH3GL1
TICAM1	MT-ND4	TICAM1
LYL1	VWF	LYL1
CEBPA	GNB1	CEBPA
BAX	MT-CO1	BAX
IRF3	MT-ND6	IRF3
SLC13A3	F3	SLC13A3
RUNX1	AGT	RUNX1
BCR	CXCL8	BCR
IIAE3	MPL	IIAE3
IMD21	STAT5B	IMD21
NEP	MT-ATP6	NEP
DKCA2	MIR223	DKCA2
CAIN	THPO	CAIN
TTG2	CASP3	TTG2
LAP	PDE4D	LAP
NS	SERPINA3	NS
IIAE8	MPO	IIAE8
CAP1	LPIN1	CAP1
PAAND	ITGB3	PAAND
MKHK1	CXCL12	MKHK1
VSCM2	PON1	VSCM2
VWDP	MIR210	VWDP
ADMIO1	CCL2	ADMIO1
MVCD3	CPT2	MVCD3
ALL	ICAM1	ALL
MDS	ACTA2	MDS
SCL	BCR	SCL

OTT	CSF3	OTT
RPRGL1	BRCA2	RPRGL1
HESJAS	GLA	HESJAS
ANE1	MT-CO3	ANE1
MONOMAC	IL4	MONOMAC
CALLA	RTEL1	CALLA
MASTC	BDNF	MASTC
EST2	INS	EST2
ACS2	APOH	ACS2
ALL3	ELN	ALL3
PBGS	REN	PBGS
CAN	BAX	CAN
RHOM1	ABCB1	RHOM1
RHOM2	HTRA1	RHOM2
RPRGL2	APOB	RPRGL2
CLTH	FGA	CLTH
PLZF	IL1RN	PLZF
UPS	EDN1	UPS
THC5	CALR	THC5
RASK2	HIF1A	RASK2
FTDALS4	MEFV	FTDALS4
LAP1	NOTCH1	LAP1
FMF	AKT1	FMF
RSTS1	HP	RSTS1
FAA4	HLA-DRB1	FAA4
BDPLT1	ELANE	BDPLT1
HIES	MYC	HIES
ACE1	CSF2	ACE1
IIAE6	F7	IIAE6
ARLIAK	PLG	ARLIAK
AML1	LPA	AML1
PHL	CDKN2B-AS1	PHL
MRD42	MIR320A	MRD42
TCL5	LMNA	TCL5
IIAE4	PLA2G7	IIAE4
SPEN	IFNG	SPEN
THPH2	LPL	THPH2
TBRS	MT-CO2	TBRS
MTPA	CXCR4	MTPA
NUP358	MB	NUP358
DCML	SETD2	DCML
CD10	ANXA5	CD10
BTL	ALOX5	BTL
PBT	HLA-B	PBT
TCS1	APP	TCS1
FACL6	MAPT	FACL6
MAR	RNF213	MAR

D6S231E	TTR	D6S231E
NBS1	PIK3C2A	NBS1
THCYT3	SELE	THCYT3
BSAP	PTPN11	BSAP
ALADH	FIP1L1	ALADH
MRD58	CYCS	MRD58
D9S46E	CDKN2B	D9S46E
RBTN1	ALDH2	RBTN1
RBTNL1	WT1	RBTNL1
THPH1	PTGS2	THPH1
IIAE1	EPO	IIAE1
CALM	EPOR	CALM
ZNF145	COL4A2	ZNF145
PBGD	IL18	PBGD
TEL	CREB1	TEL
KRAS2	LOX	KRAS2
NAK	TGFB1	NAK
FLAP	PECAM1	FLAP
CD40BP	GUCY1A1	CD40BP
MYL	TLR3	MYL
MEF	SMAD4	MEF
CBP	ADORA2A	CBP
AAT4	IL2	AAT4
PEBP2B	ADM	PEBP2B
BSS	CREBBP	BSS
GHISID2	LCN2	GHISID2
APRF	G6PD	APRF
DCP1	ITGA2B	DCP1
EEN	ESR1	EEN
TRIF	SOD2	TRIF
CEBP	VHL	CEBP
IIAE7	PPARG	IIAE7
NADC3	IL3	NADC3
CBFA2	PF4	CBFA2
CML	SH2B3	CML
CMM9	PTGIS	CMM9
OES	TGFB2	OES
SCA43	ATM	SCA43
PFBMFT1	P2RY12	PFBMFT1
RALD	MYLK	RALD
IIAE5	ADIPOQ	IIAE5
CMT2T	HFE	CMT2T
DKCB4	EDNRA	DKCB4
IIAE9	C3	IIAE9
CFC2	PBX1	CFC2
CRAF1	OLR1	CRAF1
BDPLT3	ENO2	BDPLT3

ICH	PRKAR1A	ICH
	HBB	NOTCH3
	CSF3R	TP53
	SERPINF2	MT-TL1
	CD14	IL6
	PRKG1	MTHFR
	ADRB2	FBN1
	AVP	CRP
	TNNI3	TNF
	HMGB1	LDLR
	CD34	PIK3CA
	MIR15A	PRKCH
	PDGFRA	KMT2A
	TNNT2	TET2
	GPT	IL1B
	VCAM1	ENG
	MT-CYB	APOE
	NF1	INSL6
	GSTM1	IL10
	IL17A	ACSL4
	MALAT1	NSD1
	MAPK3	MT-ND1
	HMGCR	PLAT
	CD40LG	SERPINE1
	CFH	MIR126
	PON2	COL4A1
	ANGPT1	IDH1
	CYP2C19	MMP9
	TNFRSF1A	MIR155
	APOA1	ACTB
	VKORC1	NPPB
	U2AF1	CDKN2A
	AQP4	ADAMTS13
	CTLA4	GATA1
	F10	MT-ND5
	CCL3	SELP
	MDM2	VEGFA
	MIR16-1	NPPA
	SEPTIN9	IDH2
	MIR21	AGTR1
	MBP	SERPINC1
	SIRT1	ALB
	FGB	THBD
	CCND1	ABL1
	HMOX1	PTEN
	COL3A1	CST3
	IGF1	ADA2

KNG1	TLR4
IL1A	MT-ND4
SMAD3	VWF
GATA3	MT-CO1
CASP1	MT-ND6
CASP9	F3
BCL2	AGT
HRAS	CXCL8
CCR5	MPL
LTA	MT-ATP6
SOD1	MIR223
TRAF3	THPO
TGFBR1	CASP3
NRAS	PDE4D
EDNRB	SERPINA3
TGFBR2	MPO
CYP3A5	ITGB3
FGF2	CXCL12
HSPA4	PON1
ASXL1	MIR210
PROZ	CCL2
CALCA	ICAM1
IRF2BP2	ACTA2
GSR	CSF3
ABCA1	BRCA2
NOS2	GLA
FLT1	MT-CO3
NFE2L2	IL4
FAS	RTEL1
ITGAM	BDNF
CP	INS
MME	APOH
PPBP	ELN
XIAP	REN
POLG	ABCB1
PPOX	HTRA1
MMP2	APOB
F2R	FGA
TSPO	IL1RN
PROCR	EDN1
IL6R	CALR
KITLG	HIF1A
SPP1	NOTCH1
SLC2A10	AKT1
ACVRL1	HP
MIR146A	HLA-DRB1
HADHA	ELANE

ACTA2-AS1
ABCC1
CPOX
BCL2L1
TGFB3
GDNF
JAK1
HDAC9
CTNNB1
ADORA1
CFTR
LIPC
HLA-A
ITGA2
HSPA8
STAT5A
CHEK2
TLR2
RNF213-AS1
ENPP1
CCR6
PTGS1
MMP3
FOXE3
CD4
LEP
MIF
F9
CCL11
FGFR1
SERPINA1
MBL2
LOC102723566
BMP7
RETN
PTPRC
GJA1
F13A1
FOS
IL2RA
GBA
NR3C1
ABCG2
PTX3
SLC6A4
F12
NLRP3
MYC
CSF2
F7
PLG
LPA
CDKN2B-AS1
MIR320A
LMNA
PLA2G7
IFNG
LPL
MT-CO2
CXCR4
MB
SETD2
ANXA5
ALOX5
HLA-B
APP
MAPT
RNF213
TTR
PIK3C2A
SELE
PTPN11
FIP1L1
CYCS
CDKN2B
WT1
PTGS2
EPO
EPOR
COL4A2
IL18
CREB1
LOX
TGFB1
PECAM1
GUCY1A1
TLR3
SMAD4
ADORA2A
IL2
ADM
LCN2
G6PD
ITGA2B

HLA-DQB1	ESR1
SULT1A3	SOD2
KDR	VHL
JUN	PPARG
PSEN1	IL3
CBL	PF4
CD40	SH2B3
MAP2	PTGIS
TF	TGFB2
GATA4	ATM
IFNA1	P2RY12
GP6	MYLK
NGB	ADIPOQ
AGTR2	HFE
ABCC6	EDNRA
MAPK1	C3
SLC1A2	PBX1
SELL	OLR1
PARP1	ENO2
CFI	PRKAR1A
STAT1	HBB
GNB3	CSF3R
EGF	SERPINF2
IL13	CD14
NCAM1	PRKG1
KCNE2	ADRB2
CDKN1A	AVP
NKX2-5	TNNI3
CCL5	HMGB1
MTOR	CD34
TIMP1	MIR15A
CYP3A4	PDGFRA
NES	TNNT2
SAA1	GPT
ACE2	VCAM1
TH	MT-CYB
MMACHC	NF1
HSPA1A	GSTM1
STARP1	IL17A
KCNQ1	MALAT1
XDH	MAPK3
CYP1A1	HMGCR
ANPEP	CD40LG
SERPINI1	CFH
HBA1	PON2
CASP8	ANGPT1
IL7R	CYP2C19

AOC3	TNFRSF1A
ADA	APOA1
EP300	VKORC1
ITGB2	U2AF1
MIR34A	AQP4
CSF1R	CTLA4
BRCC3	F10
LBP	CCL3
S100B	MDM2
MIAT	MIR16-1
TBXA2R	SEPTIN9
SST	MIR21
F8	MBP
TNFRSF11B	SIRT1
ACTC1	FGB
TANGO2	CCND1
AGER	HMOX1
SMAD2	COL3A1
GSTP1	IGF1
PDGFRB	KNG1
CD46	IL1A
MFAP5	SMAD3
MIR17	GATA3
SRC	CASP1
FOXP3	CASP9
HGF	BCL2
MIR9-1	HRAS
CBS	CCR5
NOS1	LTA
ADD1	SOD1
ENTPD1	TGFBR1
FASLG	NRAS
FCGR2A	EDNRB
ASS1	TGFBR2
IL1R1	CYP3A5
CETP	FGF2
SCN1A	HSPA4
GFAP	ASXL1
EZH2	PROZ
SLC22A12	CALCA
ACHE	IRF2BP2
SCN5A	GSR
MIR125A	ABCA1
ADORA3	NOS2
FN1	FLT1
TREX1	NFE2L2
NDE1	FAS

SLC19A1	ITGAM
MAT2A	CP
MMP1	PPBP
CAT	XIAP
XRCC1	POLG
CHAT	PPOX
CEBPB	MMP2
SERPIND1	F2R
CD36	TSPO
MAPK8	PROCR
RUNX2	IL6R
PRL	KITLG
MYH7	SPP1
CD8A	SLC2A10
CSF1	ACVRL1
MTAP	MIR146A
RTN4	ACTA2-AS1
IFIH1	ABCC1
SLC2A9	CPOX
CDK4	BCL2L1
DARS2	TGFB3
MAPK14	GDNF
VDR	JAK1
GDF15	HDAC9
PKD1	CTNNB1
OTC	ADORA1
MIR27A	CFTR
MEN1	LIPC
SLC17A5	HLA-A
TEK	ITGA2
NQO1	HSPA8
TERC	STAT5A
MIR142	CHEK2
ITGAL	TLR2
CXCL10	RNF213-AS1
COL4A5	ENPP1
ITIH4	CCR6
AUTS2	PTGS1
CD79A	MMP3
RYR1	FOXE3
POMC	CD4
MIR221	LEP
ITGB1	MIF
CASP2	F9
CCR2	CCL11
TNFRSF12A	FGFR1
ANGPT2	SERPINA1

CYP2C9	MBL2
NTF3	LOC102723566
FLVCR2	BMP7
HEY2	RETN
MIR451A	PTPRC
B2M	GJA1
EGFR	F13A1
CD28	FOS
DCX	IL2RA
LOC106627981	GBA
BACE1	NR3C1
H2AC18	ABCG2
GHRL	PTX3
NPY	SLC6A4
F11	F12
ECE1	NLRP3
AIF1	HLA-DQB1
CX3CR1	SULT1A3
IL5	KDR
BRAF	JUN
LGALS3	PSEN1
PDE5A	CBL
HAVCR2	CD40
PRNP	MAP2
PROM1	TF
BCL2L11	GATA4
RPL36A-HNRNPH2	IFNA1
CFB	GP6
SON	NGB
SMPD1	AGTR2
SOCS1	ABCC6
MIR181A1	MAPK1
ERCC6	SLC1A2
PROC	SELL
CD163	PARP1
PPARA	CFI
MYBPC3	STAT1
TNFSF10	GNB3
EPHX2	EGF
MTR	IL13
MIR199A1	NCAM1
SPI1	KCNE2
NGF	CDKN1A
SRFBP1	NKX2-5
CD44	CCL5
IL3RA	MTOR
MIR199B	TIMP1

BRCA1
IL15
TFRC
ZMPSTE24
MEIS1
MIRLET7B
APOC3
KCNJ5
FCGR3B
HAVCR1
MIR499A
MT-ND2
PMM2
NR1H2
IL4R
SYK
PRTN3
SMARCA4
KL

CYP3A4
NES
SAA1
ACE2
TH
MMACHC
HSPA1A
STARP1
KCNQ1
XDH
CYP1A1
ANPEP
SERPINI1
HBA1
CASP8
IL7R
AOC3
ADA
EP300
ITGB2
MIR34A
CSF1R
BRCC3
LBP
S100B
MIAT
TBXA2R
SST
F8
TNFRSF11B
ACTC1
TANGO2
AGER
SMAD2
GSTP1
PDGFRB
CD46
MFAP5
MIR17
SRC
FOXP3
HGF
MIR9-1
CBS
NOS1
ADD1
ENTPD1

FASLG
FCGR2A
ASS1
IL1R1
CETP
SCN1A
GFAP
EZH2
SLC22A12
ACHE
SCN5A
MIR125A
ADORA3
FN1
TREX1
NDE1
SLC19A1
MAT2A
MMP1
CAT
XRCC1
CHAT
CEBPB
SERPIND1
CD36
MAPK8
RUNX2
PRL
MYH7
CD8A
CSF1
MTAP
RTN4
IFIH1
SLC2A9
CDK4
DARS2
MAPK14
VDR
GDF15
PKD1
OTC
MIR27A
MEN1
SLC17A5
TEK
NQO1

TERC
MIR142
ITGAL
CXCL10
COL4A5
ITIH4
AUTS2
CD79A
RYR1
POMC
MIR221
ITGB1
CASP2
CCR2
TNFRSF12A
ANGPT2
CYP2C9
NTF3
FLVCR2
HEY2
MIR451A
B2M
EGFR
CD28
DCX
LOC106627981
BACE1
H2AC18
GHRL
NPY
F11
ECE1
AIF1
CX3CR1
IL5
BRAF
LGALS3
PDE5A
HAVCR2
PRNP
PROM1
BCL2L11
RPL36A-HNRNPH2
CFB
SON
SMPD1
SOCS1

MIR181A1
ERCC6
PROC
CD163
PPARA
MYBPC3
TNFSF10
EPHX2
MTR
MIR199A1
SPI1
NGF
SRFBP1
CD44
IL3RA
MIR199B
BRCA1
IL15
TFRC
ZMPSTE24
MEIS1
MIRLET7B
APOC3
KCNJ5
FCGR3B
HAVCR1
MIR499A
MT-ND2
PMM2
NR1H2
IL4R
SYK
PRTN3
SMARCA4
KL
