# Supplementary material for: The Neuroprotective Effect of Shenmai Injection on Oxidative Stress Injury in PC12 Cells Based on Network Pharmacology
Source: Evid Based Complement Alternat Med. 2022 May 27;2022:6969740. doi: 10.1155/2022/6969740 (PMC9166949; doi:10.1155/2022/6969740)
Supplement: Supplementary Materials — Table S1: drug targets information. Table S2: disease targets information. Table S3: intersection of drug and disease targets. [file 6969740.f1.zip › 6969740.f1/Supplementary Table S3.pdf]

SMI & AIS

VDR
HMGCR
MMP2
KDR
ABCG2
NOS2
SLC6A4
MMP3
FGFR1
PPARA
ALOX5
BACE1
CYP2C19
EGFR
NQO1
PIK3CA
FLT3
TSPO
ADORA1
ALDH2
IL2
SYK
FOS
G6PD
PDE5A
MAPK8
ADORA2A
EDNRA
F2R
TNNI3
BCL2L1
ADORA3
ABL1
PPARG
CHEK2
ANPEP
CFTR
SRC
ABCB1
NR3C1
MTOR
SERPINE1
MAPK14
TNF
CCR2
JAK1

KIT
AKT1
JAK2
NR1H2
STAT3
TNNT2
PTGS2
MIF
SIRT1
ABCC1
TGFBR1
CYP2C9
REN
CCND1
ESR1
CDK4
AGTR1
PTGS1
F3
PDE4D
PSEN1
MME
JUN
BRAF
ACHE
MMP1
ADA
CYP3A4
ITGAL
MMP9
PARP1
